# Global publication trends and research hotspots of curcumin application in tumor: A 20-year bibliometric approach

**DOI:** 10.3389/fonc.2022.1033683

**Published:** 2022-10-10

**Authors:** Jun Zhang, Yanran Huang, Jingtao Xu, Runhan Zhao, Chuang Xiong, Jiwa Habu, Yuping Wang, Xiaoji Luo

**Affiliations:** Department of Orthopedics, The First Affiliated Hospital of Chongqing Medical University, Chongqing, China

**Keywords:** curcumin, tumor, bibliometric analysis, trends, hotspots

## Abstract

Malignant tumor is a disease caused by the imbalance of cell growth and proliferation mechanism, which seriously threatens human health and life safety. However, side effects and drug resistance are the key factors that limit the efficacy of anti-tumor drugs. Therefore, it is urgent and necessary to explore and unearth natural, safe and effective chemosensitizers in tumor researches. Curcumin is the main active ingredient in Curcuma, which has anti-inflammatory, anti-inflammatory and anti-oxidation effects, and has inhibitory effects on a variety of cancers. Bibliometric analysis is a scientific and quantitative method to assess the published articles, which can help researchers to find the development trends and the research hotspots of a specific research field, providing the development of future research for researchers. This study searched the Web Science Core Collection (woscc) for publications related to curcumin and tumors from January 1, 2001 to December 31, 2021. The specific characteristics of 1707 publications were analyzed by using Microsoft Excel software, CiteSpace, Vosviewer and online analysis platform of literature metrology. China had the largest number of published articles, with 579 publications. Aggarwal BB’s articles total citations and average citations were the most. PLoS One had the largest number of publications, with 32 publications. The current research focuses on “nanoparticles”, “delivery”, “micells” and “doxorubicin”. At present, nano based drug delivery system to improve the bioavailability of curcumin and thus to treat tumors will be the focus of future research.

## Introduction

Malignant tumor is a common disease that endangers human life and health. The incidence rate and mortality of malignant tumor in the world have been on the rise (1). At present, the clinical treatment and chemotherapy effect of cancer are not good, and tumor invasion, metastasis and the generation of tumor multidrug resistance (MDR) are the main reasons for the failure of tumor treatment (2). Chemotherapy is one of the main treatment means to treat cancer at present, but cancer itself is prone to relapse and most of them end in failure. In addition, a series of toxic and side effects caused by chemotherapy also affect the quality of life of cancer patients to some degrees (3). Besides, chemotherapeutics have poor selectivity to tumor cells and normal cells. While killing or inhibiting tumor cells, they can also damage the growth of normal cells, and have a direct impact on the functions of the heart, liver, kidney and nervous system, leading to a certain toxicity to the human body (4). Therefore, it is very necessary to find low toxicity and high efficiency anti-tumor drugs in tumor treatment. Natural drugs play an important role in the prevention and treatment of cancer. They can not only prevent tumorigenesis, shrink or stabilization tumors, reduce tumor recurrence and metastasis, but also protect patients from many complications, increase the sensitivity of the body to conventional treatment, reduce side effects, improve the quality of life of patients and prolong the survival period (5, 6). Therefore, natural drugs have unique advantages and broad application prospects. Currently, various nanomaterials have been widely used to improve drug delivery potential, enhance intracellular accumulation, and provide co delivery therapy with targeted delivery of anti-tumor drugs to reduce drug resistance (7). A review conducted by Ashrafizadeh et al. systematically elaborated the application of nanoscale systems for doxorubicin delivery (7). Nanomaterials have significant advantages to overcome the problems of chemoresistance that most chemotherapeutic drugs face with. Siddharth et al. developed chitosan-dextran sulfate-coated PLGA-PVA nanoparticles for doxorubicin delivery to explore the efficacy of the nanoparticles in preventing the progression of doxorubicin-resistant cancer cells. The results demonstrated that this drug delivery system could induce cytotoxicity against breast cancer cells (MCF-7 cells) (8).

**Figure 1:**
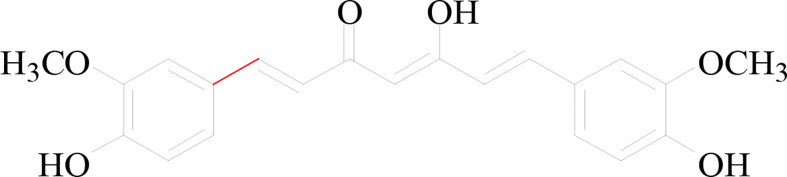
The chemical structure of curcumin. This study aimed to use bibliometric methods and visual analysis to identify research trends and potential hot spots of the application of curcumin in tumor by comprehensive analysis of relevant information of publications published worldwide during the last 20 years. What’s more, we also excavated the contents that need further researches, with a view to providing reference for clinical rational drug use.

Curcumin is a diketone compound, mainly derived from the rhizome of Curcuma, which can be used as food pigment and condiment. It was found about two centuries ago. It tastes slightly bitter, peppery, and smells like yellow mustard (9). The chemical structure of curcumin was shown in **Figure 1**. Current studies have found that curcumin has various pharmacological effects such as anti-cancer, anti-inflammatory, anti-oxidation, prevention of Alzheimer’s disease and inhibition of atherosclerosis (10–12). Curcumin in particular has great potential in the treatment of chronic diseases. Curcumin has significant therapeutic effects on gastrointestinal system diseases, nervous system diseases, diabetes and cardiovascular diseases (13). In addition, previous studies have found that curcumin has obvious inhibitory effects on a variety of tumors, mainly through inhibiting cell proliferation, inducing apoptosis, inhibiting tumor invasion and other mechanisms. Wang et al. found that curcumin could inhibit the growth of osteosarcoma cells under hypoxia by inhibiting Notch1 Signaling (14). Bai et al. found that curcumin could down regulate the expression of BCLAF1, inhibit the activation of PI3K/Akt/GSK-3β pathway, and trigger mitochondrial apoptosis in hepatoma cells (15). Wang et al. found that curcumin was capable of improving improve the sensitivity of non-small cell lung cancer to cisplatin through the endoplasmic reticulum stress pathway and could be used as one of the molecular targets to overcome cisplatin resistance (16). In recent years, different types of nanomaterials have been introduced with the capability of being utilized as carriers for delivering curcumin, including zein, chitosan, starch, silk, alginate, and so on. For example, Song et al. loaded curcumin into the magnetic silk fibroin core-shell nanoparticles for cancer targeting. This delivery system demonstrated high cellular uptake against triple negative breast cancer MDA-MB-231 cells (17). In addition, Crivelli et al. found that curcumin encapsulated in silk fibroin nanoparticles could enhance cell viability, anti-inflammatory and antioxidant activities *in vitro* (18). Leila et al. found that the co-delivery of curcumin plus Bcl-2 siRNA with the PLGA-PEI nanosystem could be a synergistic drug carrier against breast cancer cells (19). In conclusion, curcumin can exhibit anti-cancer ability by targeting different cell signaling pathways, including growth factors, cytokines, transcription factors and genes regulating cell proliferation and apoptosis. Bibliometrics is an interdisciplinary subject that evaluates and predicts the current situation and development trend of science and technology by using the measurement methods of mathematics and statistics, taking the literature system and literature metrology characteristics as the research object (20). Bibliometric evaluation objectively evaluates the development status and level of a certain field by analyzing and studying the quantity and quality of scientific literature output in this field (21). For the research in the target field, the method of bibliometrics can help researchers quickly sort out the information context in the field, including the annual publishing trend of the literature, the law of the publishing institution or journal, and popular research (22, 23).

## Materials and methods

### Search strategy

**Figure 2:**
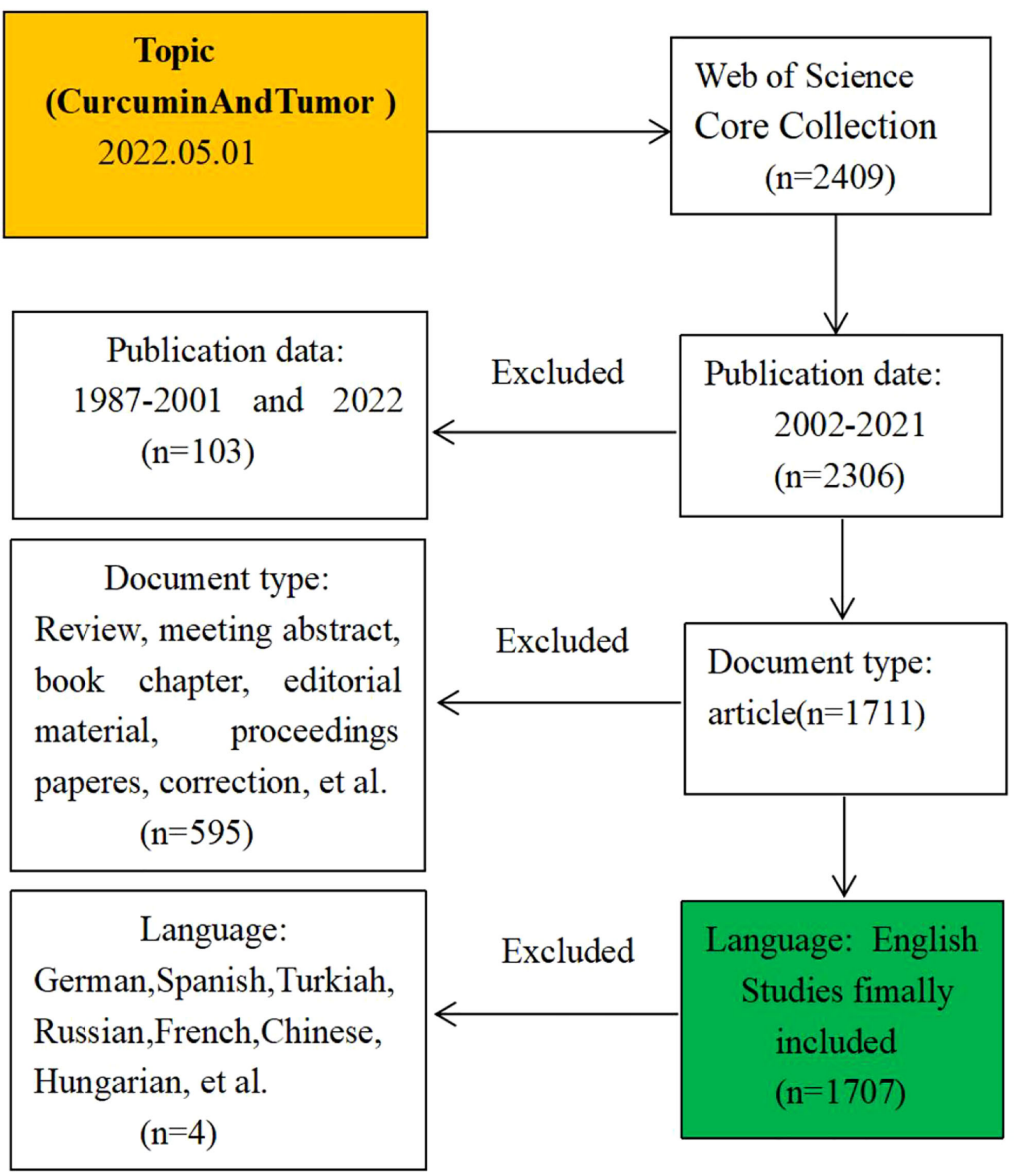
The inclusion criteria for paper selection.

This study extracted curcumin and tumor related data from the Web of Science Core Collection (WOSCC) database from January 1, 2001 to December 31, 2021. The retrieval method was shown in **Figure 2**. The search formula was TS= (curcumin or “Turmeric Yellow” or “Yellow Turmeric” or “Curcumin Phytosome” or “Phytosome Curcumin” or “Diferuloylmethane” or “Mervia”) and TS=(Tumor or Neoplasm or Tumors or Neoplasia or Neoplasias or Cancer or Cancers or “Malignant Neoplasm” or Malignancy or Malignancies or “Malignant Neoplasms” or “Neoplasm, Malignant” or “Neoplasms, Malignant” or “Benign Neoplasms” or “Benign Neoplasm” or “Neoplasms Benign” or “Neoplasm Benign”).

### Data collection

Extract Extracting complete records and cited references from the retrieved documents for bibliometric analysis, such as title, publication years, author, nationality, author organization, published journals, abstracts, keywords, total number of publications, total number of citations, average citations of each item and H index. Two authors (ZJ and HYR) independently extracted data from selected publications. If the difference remained unresolved, the results will be mediated by the third author (XJT).

### Data analysis and visualization

In this study, Microsoft Excel software, Vosviewer and CiteSpace were used to conduct cooperation among countries/regions and organizations, including literature coupling analysis, co-author analysis, reference co-citation analysis and keyword co-occurrence analysis.

## Results

### Global publication outputs and citations

**Figure 3:**
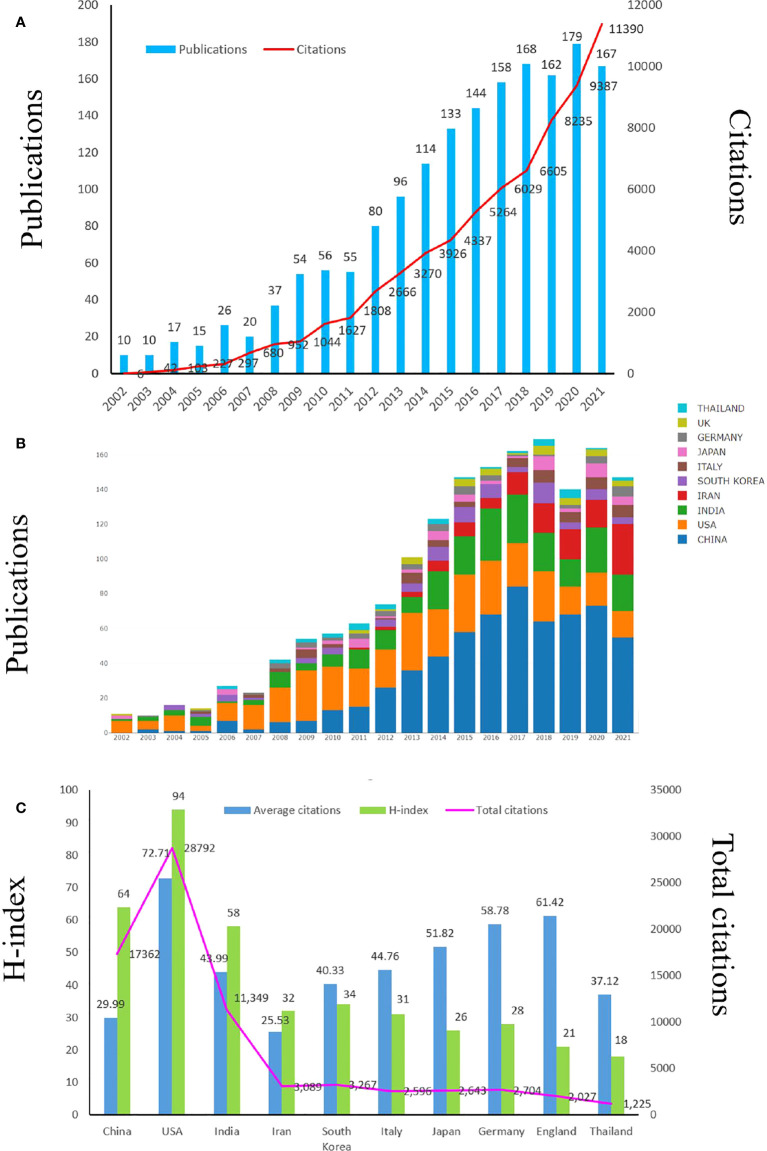
Global trends in publications about Curcumin and tumor. **(A)** The single-year publication numbers over the past 20 years. **(B)** The annual worldwide publication output. **(C)** H-index, citations per article, and total number of citations for the top 10 country/regions.

We retrieved 2409 articles related to “curcumin and tumor” from the WOSCC. According to the exclusion criteria of publication time, type and language, there were 1707 articles that could be analyzed in the next step. The temporal distribution of curcumin and tumor related publications was shown in **Figure 3A**. In the past two decades, the overall trend has been on the rise. The number of papers published each year has steadily increased, and the number of papers published in 2020 was the largest, indicating that curcumin has gradually attracted attention in the field of cancer research.

### Distribution of countries/regions

In the past 20 years, many countries in the world have published curcumin and tumor related publications. The top ten countries were shown in **Table 1**. Among them, China was the country with the largest number of relevant publications, with a total of 579 publications, accounting for 33.9%. Followed by the United States (n = 396 publications, 23.19%), followed by India (n = 258 publications,15.11%). The top ten countries in terms of total publications were Iran (n = 121, 7.08%), South Korea (n = 81, 4.74%), Italy (n = 58, 7.08%), Japan (n = 51, 2.98%), Germany (n = 46, 2.69%), England (n = 33, 1.93%) and Thailand (n = 33, 1.93%). Although the number of publications in China ranked first, the total citations was only 17362, only lower than that in the United States. Chinese publication average citations was only 29.99, far lower than other countries in the top 10, except Iran. Although the number of publications in the United States ranked second, both total citations and average citations ranked first, far higher than other countries. This demonstrated that the quality of publications in the United States was high and had certain reference value. H index is a mixed quantitative index, which can be used to evaluate the number and level of academic output of researchers. American researchers had the highest H index, indicating that their papers had great influence (**Figure 3C**). After 2013, China’s annual total publications accounted for the majority of the global total. The number of papers published by the top ten countries in total publications from 2002 to 2021 was shown in **Figure 3B**.

**Table 1:** The top 10 countries that contributed publications on the curcumin.

Rank	Country	Total publications	Total citations	Average citations	H-index
1	China	579	17362	29.99	64
2	USA	396	28792	72.71	94
3	India	258	11349	43.99	58
4	Iran	121	3089	25.53	32
5	South Korea	81	3267	40.33	34
6	Italy	58	2596	44.76	31
7	Japan	51	2643	51.82	26
8	Germany	46	2704	58.78	28
9	England	33	2027	61.42	21
10	Thailand	33	1225	37.12	18

**Figure 4:**
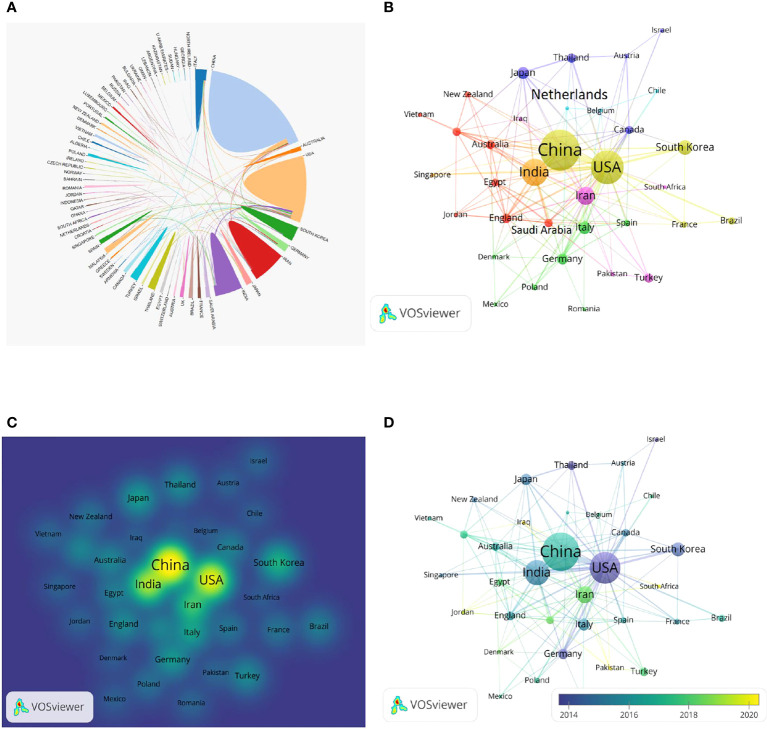
Overview of national publications. **(A)** Cooperation among different countries. **(B–D)** The collaboration network of countries/regions.

We analyzed the importance of countries in the visualization of collaborative networks using the online bibliometric analysis platform (**Figure 4A**). China had more active influence than any other countries, followed by the United States, Iran, India and South Korea. China and the United States had the closest cooperation, and the United States established cooperative relations with most countries in the world. We performed visual analysis with VOSviewer software (**Figure 4B**). The number of publications in each country could be directly reflected from the density map (**Figure 4C**). The publications of the United States and South Korea were mainly concentrated around 2014, while those of China, India and Iran were mainly concentrated after 2016 (**Figure 4D**).

### Distribution of institutions

**Figure 5:**
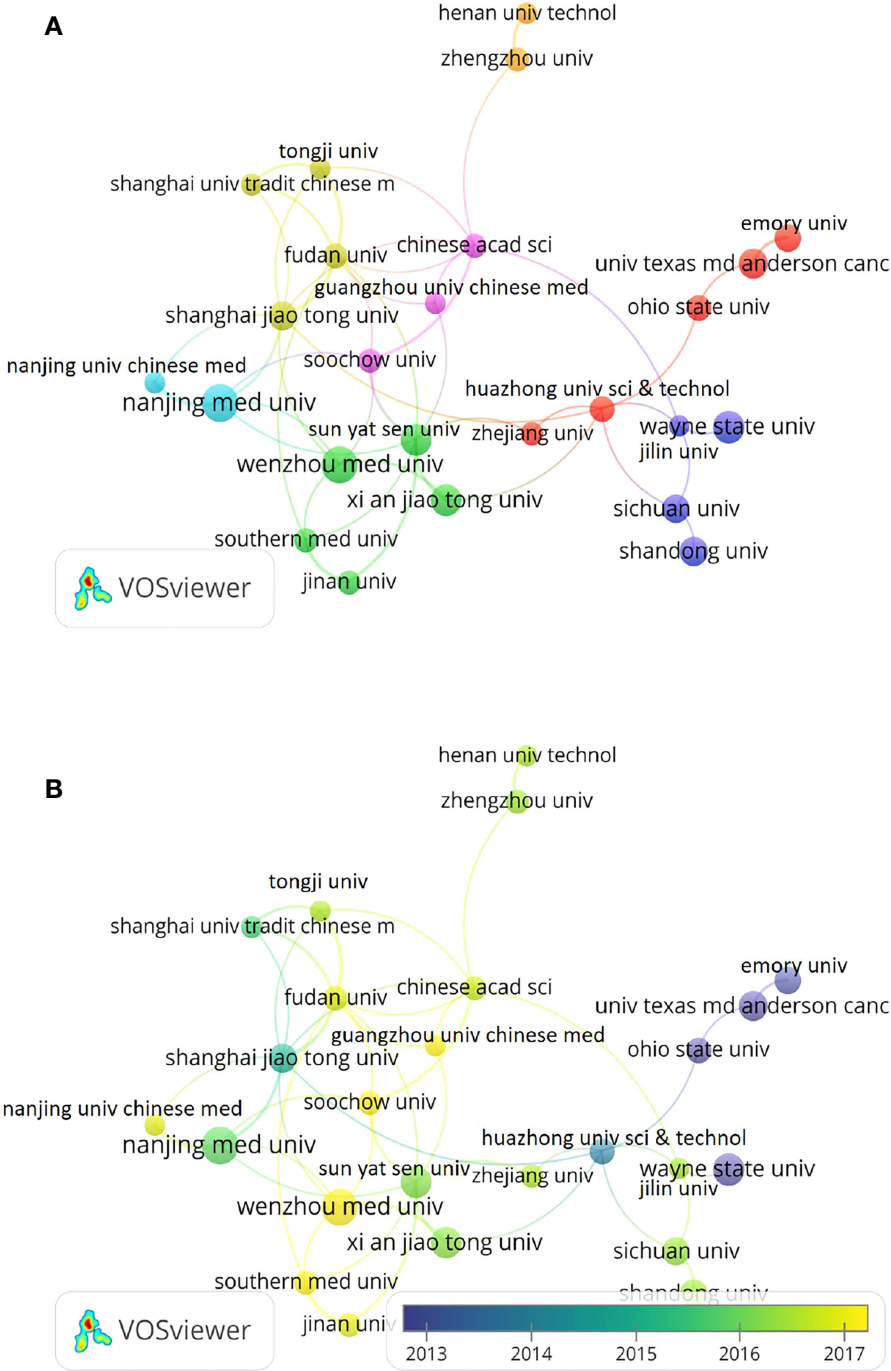
Cooperation networks between institutions based on VOSviewer. **(A)** Cooperation relationship chart of various institutions with network visualization. **(B)** Cooperation time chart of various institutions with network visualization.

Wenzhou Medical University was the institution that publishes the most research on Curcumin and tumor in the world, with a total of 36 articles. Other institutions with the top 10 productivity were Nanjing Medical University, China Med University, Islamic Azad University, Rutgers State University, tarbiat Modares University, Wayne State University, Xian Jiangtong University, Sun Yat sen University, Tabriz University Med SCI, as shown in **Table 2**. In addition, Nanjing Medical University had the highest total citations (1448), average citations (42.59) and H-index (23), indicating that the publications of this institution were highly recognized. The cooperation relationship between institutions was shown in **Figure 5A**, and the cooperation time chart of each institution from 2002 to 2021 was shown in **Figure 5B**.

**Table 2:** The top 10 institutions that contributed publications on curcumin.

Rank	Institutions	Country	Total publications	Total citations	Average citations	H-index
1	Wenzhou Medical University	China	36	811	22.53	18
2	Nanjing Medical University	China	34	1448	42.59	23
3	China Med University	China	24	1240	51.67	18
4	Islamic Azad University	Iran	26	308	11.85	11
5	Rutgers State University	USA	27	1240	45.93	19
6	Tarbiat Modares University	Iran	26	675	25.96	15
7	Wayne State University	USA	25	2626	105.04	23
8	Xian jiangtong University	China	24	917	38.21	18
9	Sun yat sen University	China	23	973	42.3	17
10	Tabriz University Med Sci	Iran	23	642	27.91	14

### Authors and co-cited authors

**Figure 6:**
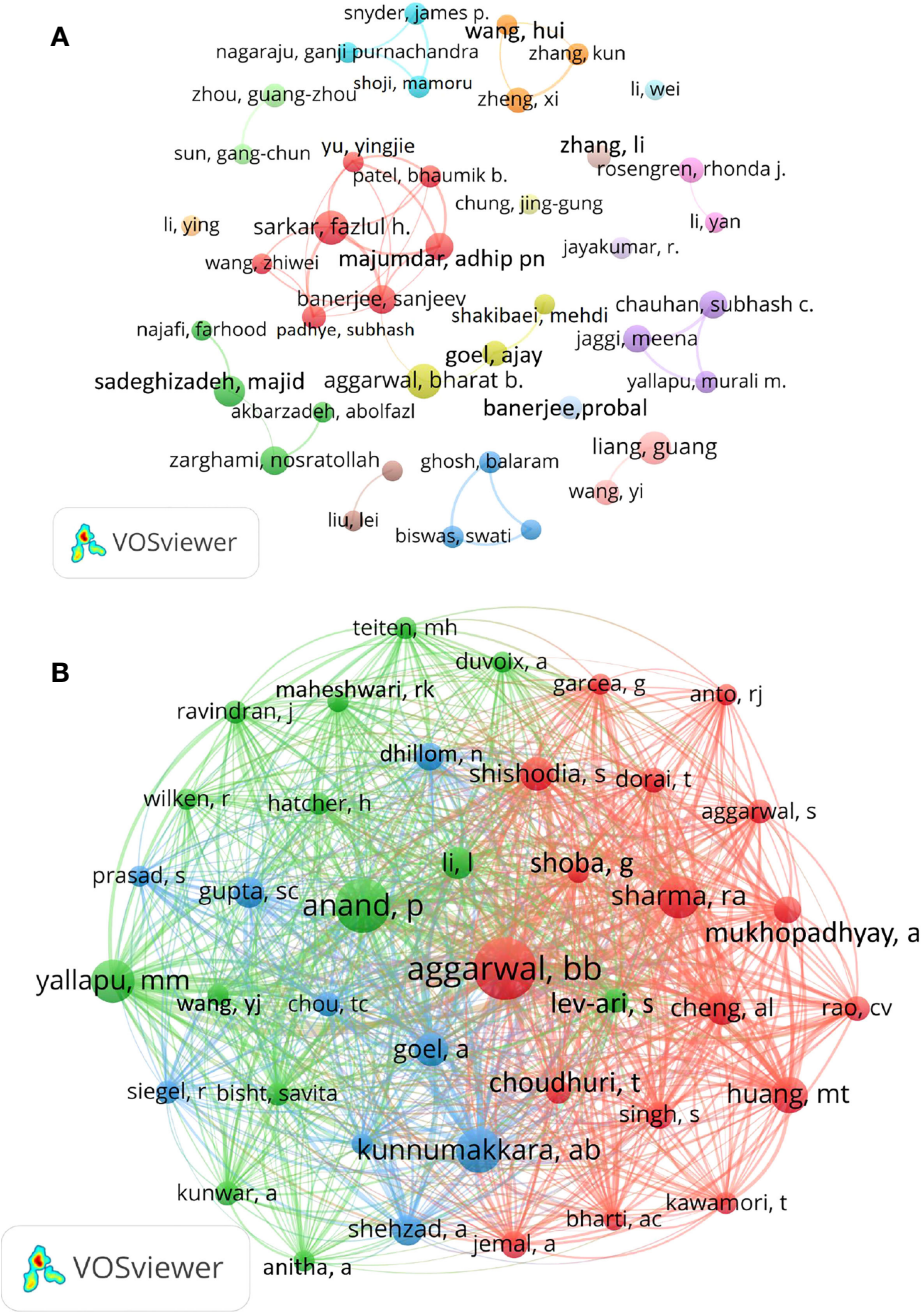
Visualization of active authors analysis. **(A)** Network visualization analysis of author collaboration. **(B)** Network visualization map of co-cited authors of the articles.

The top 10 most productive and the top 10 most co-cited authors were listed in **Table 3**, and visual analysis was performed with VOSviewer software (**Figures 6A, B**). Among them, Aggarwal BB and Banerjee S were the authors with the largest number of curcumin and tumor related publications, 20 publications respectively. However, Aggarwal BB had the highest total citations, with average citations of 208.4 and H-index of 19, indicating that he made great achievements in curcumin and tumor related researches and had certain influence. The strongest collaboration was between Fazlul H Sarkar, Adhip P N Majumdar, Sanjeev Banerjee, Yu yingjie, Bhaumik B Patel, and Wang zhiwei.

**Table 3:** The top 10 authors and co-cited authors that contributed publications on curcumin.

Authors	Total publications	Total citations	Average citations	H-index	Co-cited author	Citations
Aggarwal BB	20	4168	208.4	19	Aggarwal BB	661
Banerjee S	20	2554	127.7	18	Anand P	502
Sarkar FH	18	2123	117.94	17	Kunnumakkara AB	343
Liang Guang	17	483	28.41	13	Yallapu MM	312
Sadeghizadeh M	15	382	25.47	9	Sharma RA	297
Zhang Li	15	345	23	10	Huang MT	225
Zarghami N	13	525	40.38	11	Goel A	194
Chauhan SC	12	1758	146.5	12	Shishodia S	190
Wang Yi	12	780	37.14	15	Cheng AL	181
Chauhan SC	12	1758	146.5	12	Gupta SC	165

### Journals and co-cited journals

**Figure 7:**
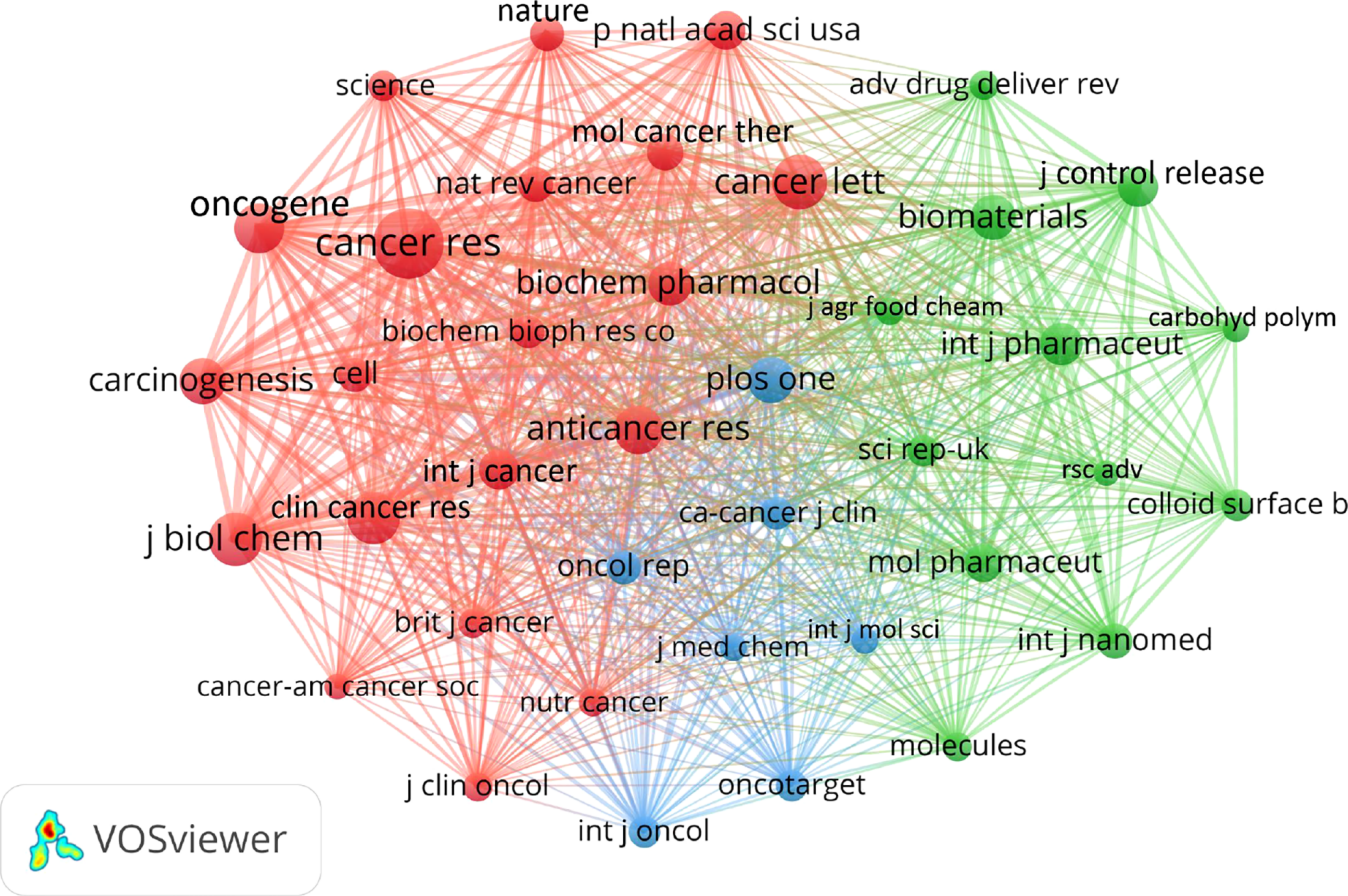
Co-cited journals of curcumin and tumor articles were published by vosviewer analysis.

**Figure 8:**
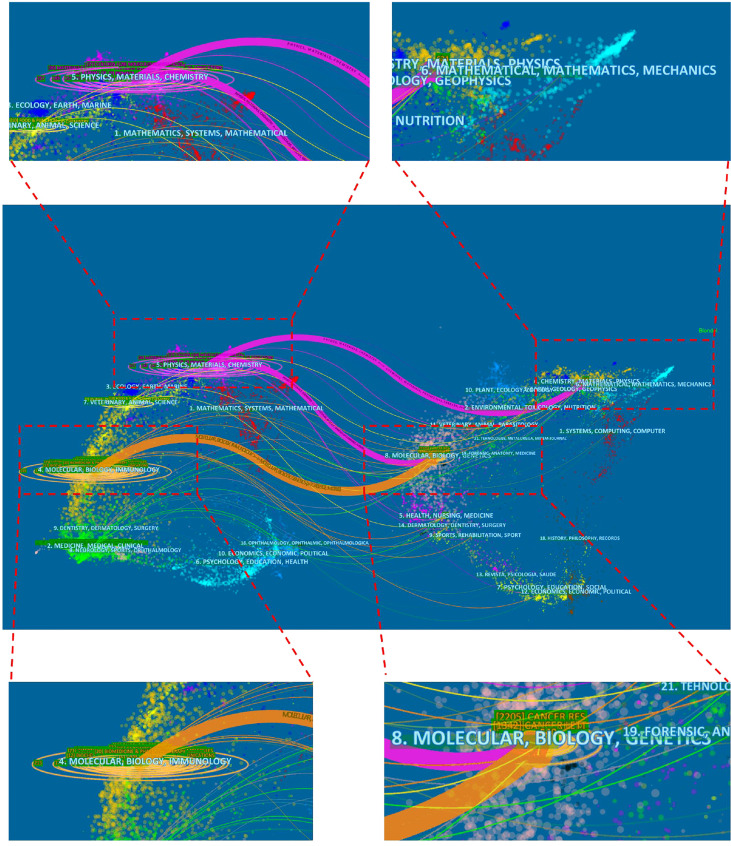
The dual-map overlay of articles citing on curcumin and tumor research. (The left side were the citing journal, the right side were the cited journal, and the line path represented the citation relationship).

In terms of journals, the top ten journals that published articles on curcumin and tumors were shown in **Table 4**. Among them, *Plos One* published the most articles (n=35, IF=3.240), followed by *Oncology Reports* (n=32, IF=3.906), *Molecules* (n=28, IF=4.411), *Anticancer Research* (n=27, IF=2.480), *Colloids And Surfaces B Biointerfaces* (n=27, IF=5.268). VOSviewer analysis demonstrated that Cancer Research, Cancer Letters and Journal of Biological Chemistry ranked among the top three in the co-citation rate (**Figure 7**). **Figure 8** revealed a dual map overlay of research topics between citing and cited journals in the field of curcumin and tumor research. The purple-red path indicated that studies published in “*Physics, Materials, Chemistry*” journals tend to be cited primarily in *“Mathematical, Mathematics, Mechanics, Geophysics”.* Orange paths indicated that studies published in *“Molecular, Biology, Immunology”* journals tend to be cited primarily in *“Molecular, Biology, Genetics”* (**Figure 8**).

**Table 4:** The top 10 journals and co-cited journals that published articles on curcumin.

Journal	Articles Counts	Country	JCR	IF	Co-cited Journal	Cocitation	JCR	IF
**Plos One**	35	USA	Q2	3.240	Cancer Research	2205	Q1	12.701
Oncology Reports	32	Greece	Q2	3.906	Cancer letters	1342	Q1	8.679
Molecules	28	Switzerland	Q2	4.411	Journal of Biological Chemistry	1271	–	5.157
Anticancer Research	27	Greece	Q4	2.480	Clinical Cancer Research	1202	Q1	12.531
Colloids And Surfaces B Biointerfaces	27	Netherlands	Q1	5.268	Oncogene	1130	Q1	9.867
Carcinogenesis	25	England	Q2	4.944	Anticancer Research	1067	Q4	2.480
International Journal of Nanomedicine	25	New Zealand	Q2	6.400	Carcinogenesis	950	Q2	4.944
Scientific Reports	25	England	Q1	4.379	Plos One	931	Q2	3.240
Nutrition and Cancer an International Journal	23	USA	Q4	2.900	Biomaterials	896	Q1	12.479
International Journal of Oncology	22	Greece	Q2	5.650	Biochemical Pharmacology	851	Q1	5.858

### Co-cited references

**Figure 9:**
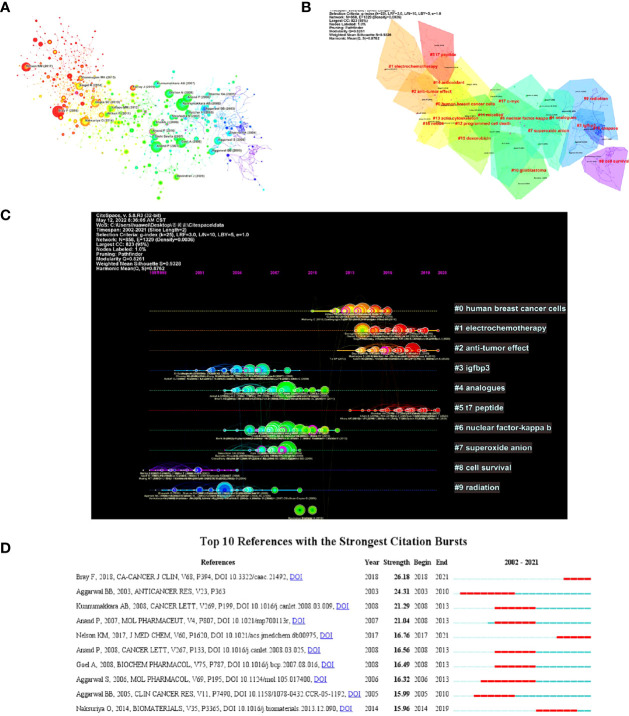
Cocitation analysis of references. **(A)** Network visualization analysis of co-cited References. **(B)** Cluster Analysis of Co-cited References. **(C)** Timeline view of references in the field of curcumin and tumor. **(D)** The top 10 references with the strongest citation bursts on curcumin and tumor research.

By analyzing the key nodes and clusters in the reference co-citation network, we can reveal the core themes of a certain research field, and understand the knowledge base and the evolution of research fronts in this field (22). The co-cited reference network was shown in **Figure 9A**, where the cited references were marked, and the specific information was shown in **Table 5**. The literature with the most co-cited references was “Curcumin inhibits proliferation, invasion, angiogenesis and metastasis of different cancers through interaction with multiple cell signaling proteins” published by Kunnumakkara AB in Cancer Lett in 2008 (cited 57 times in total). The nine categories of documents and their references generated by Citespace software were shown in **Figures 9B, C**. Nine distinct clusters were labeled “#0 human breast cancer”, “#1 electrochemoth therapy”, “#2 anti-tumor effect”, “#3 IGFBP3”, “#4 analogues”, “#5 T7 peptide”“, “#6 nuclear factor-kappa”, “#7 superoxide anion” and “#8 cell survival”. The top 10 references with the strongest citation bursts were shown in **Figure 9D**, indicating their importance in curcumin and tumor-related fields.

**Table 5:** The top 10 most co-cited references related to curcumin.

Author	Citations	Title	Journal	Year
Kunnumakkara AB	57	Curcumin inhibits proliferation, invasion, angiogenesis and metastasis of different cancers through interaction with multiple cell signaling proteins	Cancer Lett	2008
Preetha Anand	52	Bioavailability of curcumin: problems and promises	Mol Pharm	2007
Goel A	48	Curcumin as “Curecumin”: from kitchen to clinic	Biochem Pharmacol	2008
Ornchuma Naksuriya	44	Curcumin nanoformulations: a review of pharmaceutical properties and preclinical studies and clinical data related to cancer treatment	Biomaterials	2014
Nelson KM	44	The Essential Medicinal Chemistry of Curcumin	J Med Chem	2017
Anand P	43	Curcumin and cancer: an “old-age” disease with an “age-old” solution	Cancer Lett	2008
Yallapu MM	42	Curcumin nanoformulations: a future nanomedicine for cancer	Drug Discov Today	2012
Navneet Dhillon	40	Phase II trial of curcumin in patients with advanced pancreatic cancer	Clin Cancer Res	2008
Gupta SC	38	Therapeutic roles of curcumin: lessons learned from clinical trials	AAPS J	2013
Aggarwal S	37	Curcumin (diferuloylmethane) down-regulates expression of cell proliferation and antiapoptotic and metastatic gene products through suppression of IkappaBalpha kinase and Akt activation	Mol Pharmacol	2006

### Analysis of keywords

**Figure 10:**
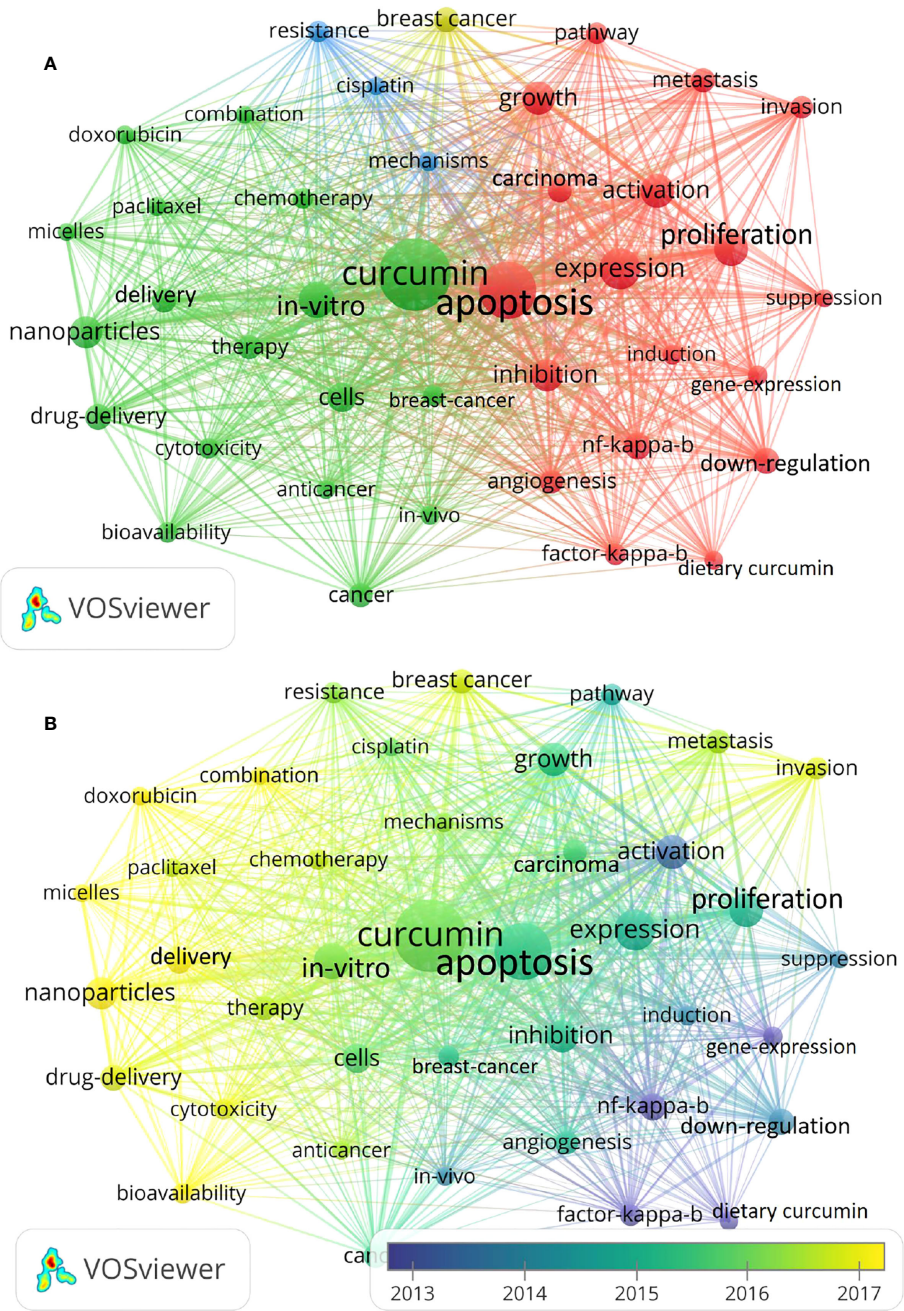
Visualization of keyword analysis. **(A)** The network map of keywords. **(B)** Evolution of keyword frequency.

Keyword co-occurrence analysis can reflect the research hotspot in a field. We used VOSviewer software for visual analysis, and the keywords with the highest frequency were “curcumin, apoptosis and expression” (**Figure 10A**). We also divided the color of keywords according to the average publication year by VOSviewer (**Figure 10B**). Blue keywords indicated that they appeared earlier than the yellow keywords. In the early years, “NF-KB”, “factor-kappa” and “gene expression” were the main topics. In addition, many other keywords appeared in recent years, such as “nanoparticles”, “delivery”, “micells” and “doxorubicin”, which indicated that the current hot or cutting-edge research in these fields related to curcumin and tumors.

## Discussion

In the current cancer treatment, tumor cells have developed invasion, metastasis and drug resistance after treatment with many chemical drugs. This trend makes the effect of cancer treatment ineffective and the prognosis of surgery poor (4). In recent years, it has been reported that natural drugs can not only inhibit tumor invasion, metastasis and reverse drug resistance, but also inhibit the invasion and metastasis of tumor cells in combination with chemical drugs, which are capable of reverse drug resistance. At present, the combination of Chinese and Western medicine can significantly improve the prognosis of patients with malignant tumors. Curcumin is a multi-target Chinese medicine monomer compound, which can not only regulate the functional differentiation of immune cells and the expression of cytokines in the tumor microenvironment, but also can be used as a combination chemotherapy drug for the treatment of tumors. It has great potential in the field of tumor treatments (24). In this study, we analyzed a total of 1707 literatures from January 1, 2001 to December 31, 2021, and the number of publications on curcumin and tumor related research increased year by year, indicating that curcumin might be the research hotspot of natural drugs for tumor treatments. China was the country with the largest number of relevant publications in the last 20 years, but average citations was only 29.99, which was far lower than other countries in the number of publications, indicating that the quality of China’s publications still needed to be improved. The United States and China had the closest cooperative relations, and the United States has established good cooperative relations with most countries in the world. In terms of institutions, Wenzhou Medical University was the institution that published the most researches on curcumin and tumor in the world. In their latest researches, they designed two dicarbonyl curcumin analogues with improved stability, and screened their anti-tumor activities, showing excellent *in vivo* and *in vitro* lung cancer activity (25). We analyzed the most influential authors, and the results showed that Aggarwal BB and Banerjee s were the authors with the largest number of curcumin and tumor related publications, 20 respectively. However, in comparison, Aggarwal BB had the highest total citations, with average citations of 208.4 and H-index of 19. He was better in overall influence. His latest research demonstrated that Calebin A, a derivative of curcumin, was a new drug with anticancer potential. Calebin A has an anti-cancer capability on TNF-β-induced malignities through inhibitory targeting of NF-B activation in the cytoplasm, as well as by suppressing the binding of p65-NF-B to DNA (26). In addition, *PLoS One* was the journal that published the most articles related to curcumin and tumor, and most publications focused on the research of molecular mechanism. Among them, the literature with the largest number of co-cited references was Kunnumakkara AB’s “Curcumin inhibits promotion, invasion, angiogenesis and metatasis of different cancers through interaction with multiple cell signaling proteins” published in Cancer Letter in 2008. The article summarized the role of curcumin in regulating multiple cellular pathways, and summarized the results of several phase I and phase II clinical trials, indicating that curcumin was quite safe and may show therapeutic efficacy, reflecting the importance of curcumin in clinical cancer treatment (27). It is worth noting that no matter from the analysis of the cooperative relationship map between research institutions or the cooperative relationship map of the authors, the density of cooperative relationships is not large, there are few contacts between them and the interactivity is poor. Therefore, strengthening academic cooperation between different countries and institutions and forming an academic community is an important aspect to promote the clinical and academic research development of this topic in the future.

Through the co-occurrence analysis, cluster analysis, time line view analysis and emergence analysis of the research literature and its keywords, we can conclude that the most studied cancer of curcumin is breast cancer. Curcumin exerts its anticancer effect through a complex molecular signaling network, involving proliferation, estrogen receptor (ER) and human epidermal growth factor receptor 2 (HER2) pathways. Experimental evidence shows that curcumin also regulates apoptosis and cell phase related genes and microRNAs in breast cancer cells (28, 29). Although curcumin has a broad application prospect, its poor solubility, low bioavailability, poor permeability and high metabolic activity hinder its development as an effective anticancer drug. Therefore, the keywords appearing in recent years, such as “nanoparticles”, “delivery” and “micells”, indicate the hot or frontier research of curcumin in the field of cancer. According to recent studies, the nano preparations of curcumin mainly include nanoparticles, liposomes, micelles, polymers, nano gel, etc. They can overcome the biological defects of curcumin and improve its stability and cell bioavailability *in vitro* and *in vivo* (30, 31). Although there are many shortcomings, at present, curcumin nanoparticles still have good development potential and application prospects in tumor treatment.

The therapeutic use of curcumin was first reported in 1748, but the first article reporting its use in human disease was published by Oppenheimer in 1937 (32). The safety, tolerance and non-toxic of high-dose curcumin have been fully confirmed by human clinical trials in the past decades. So far, more than 230 clinical trials have been successfully carried out to understand the pharmacological effects of curcumin in the human system (33). Curcumin can effectively alleviate the signs and symptoms of diseases, reduce the level of biomarkers, improve the quality of life, slow down the progress, and prevent the recurrence of patients’ diseases. In 2001, a phase I study reported that the histology of precancerous lesions in patients with recently resected bladder cancer, leukoplakia, intestinal metaplasia of the stomach, uterine cervical intraepithelial neoplasm and Bowen’s disease was significantly improved after curcumin treatment. At the same time, the study also showed that curcumin could be administered orally for 3 months, and the amount of non-toxic agent to human body could reach 8000 mg/day (34). Wolf indicated that prophylactic treatment with topical curcumin may be effective for minimizing skin reactions and pain for breast cancer patients with high breast separation (35). In addition, several clinical studies have been investigating the anticancer effects of curcumin in combination with other drugs. A study has shown that the combination therapy of gemcitabine and curcumin is not only safe, but also has a certain role in improving the prognosis of patients with advanced pancreatic cancer (36). Recent clinical studies have focused on various new bioavailable curcumins, and these new protocols need to be clinically tested in randomized, placebo-controlled, multicenter trials to confirm the huge potential of curcumin in anti-cancer. In conclusion, the anti-tumor effect of curcumin and clinical trials have shown the anti-tumor effect of curcumin, revealing the clinical efficacy of anti-tumor.

## Conclusions

Through systematic bibliometric analysis and the construction of a visual map, this study summarizes and analyzed the literature publication, research topics, research hotspots and development trends of the research in this field, in order to provide a reliable reference for the research of curcumin treatment of tumors. China was the country that published the most relevant publications, and the United States ranked. first in total citations and average citations. Wenzhou Medical University is the most productive institution, and Nanjing Medical University has the highest total number of citations. Current research hotspots include “nanoparticles”, “delivery”, “micells” and “doxorubicin”.

## Data availability statement

The original contributions presented in the study are included in the article/supplementary material. Further inquiries can be directed to the corresponding author.

## Author contributions

All authors made a significant contribution to the work reported, whether that is in the conception, study design, execution, acquisition of data, analysis and interpretation, or in all these areas, took part in drafting, revising or critically reviewing the article, gave final approval of the version to be published, have agreed on the journal to which the article has been submitted, and agree to be accountable for all aspects of the work.

## Funding

This study was supported by the National Natural Science Foundation of China (NO.81873998), Doctoral student innovation project of the First Affiliated Hospital of Chongqing Medical University (CYYY-BSYJSCXXM-2002219, CYYY-BSYJSCXXM-2002215).

## Conflict of interest

The authors declare that the research was conducted in the absence of any commercial or financial relationships that could be construed as a potential conflict of interest.

## Publisher’s note

All claims expressed in this article are solely those of the authors and do not necessarily represent those of their affiliated organizations, or those of the publisher, the editors and the reviewers. Any product that may be evaluated in this article, or claim that may be made by its manufacturer, is not guaranteed or endorsed by the publisher.
